# Nitric Oxide-Releasing Bacterial Cellulose/Chitosan Crosslinked Hydrogels for the Treatment of Polymicrobial Wound Infections

**DOI:** 10.3390/pharmaceutics14010022

**Published:** 2021-12-22

**Authors:** Nurhasni Hasan, Juho Lee, Hye-Jin Ahn, Wook Ryol Hwang, Muhammad Akbar Bahar, Habibie Habibie, Muhammad Nur Amir, Subehan Lallo, Hong-Joo Son, Jin-Wook Yoo

**Affiliations:** 1 College of Pharmacy, Pusan National University, Busan 46241, Republic of Korea; nurhasni.hasan@unhas.ac.id (N.H.); jhlee2350@gmail.com (J.L.); 2 Faculty of Pharmacy, Hasanuddin University, Jl. Perintis Kemerdekaan KM 10, Makassar 90245, Indonesia; Akbarbahar@unhas.ac.id (M.A.B.); habibie@unhas.ac.id (H.H.); nuramir@unhas.ac.id (M.N.A.); subehan@unhas.ac.id (S.L.); 3 School of Mechanical and Aerospace Engineering, Gyeongsang National University, Jinju 52828, Republic of Korea; ahnhyejin@gnu.ac.kr (H.-J.A.); wrhwang@gnu.ac.kr (W.R.H.); 4 College of Natural Resources and Life Science/Life and Industry Convergence Research Institute, Pusan National University, Miryang 627706, Republic of Korea; shjoo@pusan.ac.kr

**Keywords:** polymicrobial-infected wound healing, bacterial cellulose, nitric oxide, crosslinked hydrogels, antibacterial

## Abstract

Polymicrobial wound infections are a major cause of infectious disease-related morbidity and mortality worldwide. In this study, we prepared a nitric oxide (NO)-releasing oxidized bacterial cellulose/chitosan (BC_TO_/CHI) crosslinked hydrogel to effectively treat polymicrobial wound infections. Linear polyethyleneimine diazeniumdiolate (PEI/NO) was used as the NO donor. The aldehyde group of BC_TO_ and the amine of CHI were used as crosslinked hydrogel-based materials; their high NO loading capacity and antibacterial activity on the treatment of polymicrobial-infected wounds were investigated. The blank and NO-loaded crosslinked hydrogels, namely BC_TO_-CHI and BC_TO_-CHI-PEI/NO, were characterized according to their morphologies, chemical properties, and drug loading. BC_TO_-CHI-PEI/NO exhibited sustained drug release over four days. The high NO loading of BC_TO_-CHI-PEI/NO enhanced the bactericidal efficacy against multiple bacteria compared with BC_TO_-CHI. Furthermore, compared with blank hydrogels, BC_TO_-CHI-PEI/NO has a favorable rheological property due to the addition of a polymer-based NO donor. Moreover, BC_TO_-CHI-PEI/NO significantly accelerated wound healing and re-epithelialization in a mouse model of polymicrobial-infected wounds. We also found that both crosslinked hydrogels were nontoxic to healthy mammalian fibroblast cells. Therefore, our data suggest that the BC_TO_-CHI-PEI/NO developed in this study improves the efficacy of NO in the treatment of polymicrobial wound infections.

## 1. Introduction

Acute and chronic wounds are major public health concerns worldwide. Particularly, chronic wounds colonized by bacteria have a significant impact on the increasing number of morbidity and mortality cases [1]. The vast majority of chronic wound infections are polymicrobial (i.e., caused by multiple microorganisms) in nature. Gram-negative *Pseudomonas aeruginosa*, Gram-positive *Staphylococcus aureus* (*S. aureus*), methicillin-resistant *Staphylococcus aureus* (MRSA), and *Candida* spp. fungi are the most common microorganisms that induce polymicrobial wound infections [2,3]. In the polymicrobial population, normally, several microorganisms share a niche, and coexist. As a result, the lytic enzymes and free radicals produced can affect and delay the progress of wound healing [4,5]. In addition, Pastar et al. reported that the interaction between MRSA USA300 and *Pseudomonas aeruginosa* induces the expression of USA300 virulence factors (α-hemolysin and Panton–Valentine leucocidin), resulting in an increased severity of polymicrobial wound infection [6]. Polymicrobial wound infections also pose a high risk in patients with chronic diabetic ulcers, in whom lower-limb amputation often becomes the unwanted final choice [7]. In addition, antibiotic resistance can also complicate the treatment of polymicrobial wound infections. Therefore, new treatments for polymicrobial-infected wounds, with a low risk of developing antibiotic resistance, and which have a direct impact on eradicating infectious pathogens are urgently needed.

Previously, we developed several nitric oxide (NO) delivery systems for the treatment of bacteria-/biofilm-infected wounds ranging from nanoparticles (NPs), including bacteria-targeted NPs; microparticles; ointments; films; in situ and thermoresponsive hydrogels; and NO-doped polymers [8,9,10,11,12,13,14,15,16]. In recent years, NO has emerged as a potent antibacterial, antibiofilm, and wound healing enhancing agent. Moreover, due to the diverse mechanisms of NO in killing bacteria, the possibility of NO causing bacterial resistance is minimal [17,18]. Therefore, the exploration of new NO delivery systems for the treatment of infectious wounds, especially polymicrobial wound infections, is urgently needed.

Topical application of antibiotic-based wound dressing is still favorable because of the avoidance of systemic side effects [19]. Crosslinked hydrogels are a promising wound dressing for antibiotic delivery in the treatment of polymicrobial wound infections. Hydrogels have been intensively studied for application in tissue engineering, and for the development of wound dressings for chronic wounds [20,21]. Other types of hydrogels, such as multifunctional silver NPs, have also been recently reported to accelerate healing of chronic wounds, such as planktonic- and mature bacterial biofilm-infected wounds [22,23]. Hydrogels can be prepared from polysaccharides (e.g., chitosan, alginates, cellulose, chitin, heparin), and proteins (e.g., gelatin, fibrin, silk fibroin, collagen), or synthetic polymers [24]. These materials are known for their biocompatibility, biodegradability, and similarity to the extracellular matrix; therefore, they are suitable as materials for hydrogel-based dressings [25].

Bacterial cellulose (BC) is a natural polysaccharide that is synthesized by bacteria. Its nanofiber structure and flexibility being molded into various shapes offer several benefits, especially in tissue engineering [26,27,28]. In recent years, surface modification of BC has been explored to increase its bioactivity. The surface modification of BC by introducing functional groups, such as primary hydroxy and carboxylate groups, has been studied to increase the functionalization and degradation of BC [29,30,31]. Moreover, the nanofibrous structure of BC facilitates cell migration toward the wound bed, and enhances angiogenesis [32,33]. Chitosan (CHI), a well-known polysaccharide containing 1,4-linked 2-amino-2-deoxy—D-glucose (glucosamine) and N-acetyl glucosamine, has long been used as a wound dressing and hydrogel-based material. It has been reported to increase fibroblast proliferation and collagen deposition by inducing spontaneous blood coagulation [34,35]. Combining BC and CHI in hydrogels can improve mechanical properties and rheology qualities of wound dressing materials, as both are essential macromolecules for applications in tissue engineering and wound dressing materials.

In the present work, we developed an NO-releasing BC/CHI crosslinked hydrogel for the effective treatment of polymicrobial-infected wounds. Surface properties of BC can be fine-tuned, such as by introducing an aldehyde group via the 2,2,6,6-tetramethylpiperidine-1-yl)oxyl (TEMPO) reaction. Linear polyethyleneimine diazeniumdiolate (PEI/NO), which is used as an NO donor, was crosslinked with BC. The amine group of the CHI structure was crosslinked with aldehyde from oxidized BC to increase the wound dressing properties. After evaluating their wound dressing properties, hydrogels were tested for in vitro drug release, and in vitro and in vivo bactericidal effects. Finally, in vivo wound healing activity was evaluated using an ICR mouse model of polymicrobial-infected wounds.

## 2. Materials and Methods

### 2.1. Materials

Medium-molecular weight CHI (120 cps viscosity, 85% acetylation), Avertin anesthesia component (2,2,2-tribromoethanol and *tert*-amyl alcohol/2-methyl-2-butanol), TEMPO, tetrazolium dye 3-(4,5-di-methylthiazol-2-yl)-2,5-diphenyltetrazolium bromide (MTT), citric acid, sodium bromide (NaBr, ≥99%), sodium hydroxide (NaOH, 99%), 10% sodium hypochlorite (NaClO, 99%), glucose, disodium hydrogen phosphate (Na_2_HPO_4_. 12H_2_O), dimethyl sulfoxide (DMSO), and N,N’-dicyclohexylcarbodiimide (DCC) were purchased from Sigma-Aldrich (St. Louis, MO, USA). Luria-Bertani (LB), Bacto^TM^ tryptic soy broth (TSB), yeast extract, and Bacto^TM^ peptone media were purchased from BD Biosciences (Sparks, MD, USA). Phosphate-buffered saline (PBS; 20×) was obtained from Biosesang (Seoul, Korea).Trypsin, Roswell Park Memorial Institute (RPMI) 1640 medium, penicillin-streptomycin, and fetal bovine serum (FBS) were obtained from Hyclone (Thermo Fisher Scientific Inc., Waltham, MA, USA). Linear polyethyleneimine diazenium diolates (PEI/NO) were synthesized by our group. All of the other reagents and solvents were of the highest analytical grade.

### 2.2. Preparation of Bacterial Cellulose

The bacterial strain (*Acetobacter intermedius* BC-4) was obtained from the Department of Life Science and Environmental Biochemistry, Pusan National University [36]. *Acetobacter intermedius* were grown in culture media containing glucose (2%), yeast extract (0.5%), Bacto peptone (0.5%), Na_2_HPO_4_. 12H_2_O (0.675%), and citric acid (0.115%) for 15–20 days. The pH of the medium was adjusted to six using acetic acid. The BC pellicle began to form after 7 days. BC pellicles with the appropriate thickness (10–12 μm) were then harvested by soaking in deionized water at 90 °C for 2 h. Next, the BC pellicles were immersed in a 0.5 M NaOH solution and boiled for 20 min before being washed several times with deionized water. The pellicles were then immersed in 1 wt% NaOH for two days. After rinsing with deionized water until the pH was neutral, the BC pellicles were freeze-dried for 1–2 days. The dry BC was maintained at ± 25 °C for future use.

### 2.3. Preparation of Oxidized Bacterial Cellulose via TEMPO Reaction

The carboxylic group was introduced into the BC structure by oxidation, according to a previously reported method, [37] with minor modification. Under constant stirring, BC pellicles (approximately 10 g) were put into a solution containing NaBr (1.3 g) and TEMPO (0.13 g) in deionized water. This mixture was then added to 10% NaClO solution (0.267 g) progressively, and the pH of the solution was maintained at 10.5 by using 0.5 M NaOH solution. For 30 min, the temperature was kept at 30 °C. The Oxidation was stopped by adding 40 mL of 100% ethanol. Deionized water was used to rinse the oxidized BC (BC_TO_) pellicles.

### 2.4. Preparation of Crosslinked Hydrogels

**Scheme 1:**
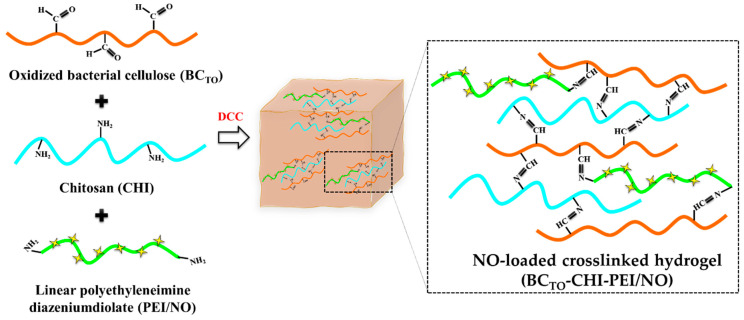
Scheme of cross-linking reaction of oxidized BC (BC_TO_) with chitosan (CHI) and linear polyethyleneimine diazeniumdiolate (PEI/NO) using N, N’-dicyclohexylcarbodiimide (DCC) as the crosslinker agent.

CHI stock solution was prepared in 100 mL of acetic acid (0.5 M). First, 2 g of CHI powder were suspended in distilled water for 25 min at 120 °C by vigorous stirring in a water bath, followed by the addition of acetic acid to obtain a 0.5 M CHI stock solution. At room temperature, two grams of oxidized BC (BC_TO_) were dispersed in 100 mL of deionized water by stirring until a homogeneous solution was obtained (BC_TO_ stock). A 1:1 ratio of stock solutions was used to prepare crosslinked BC_TO_/CHI hydrogels. To accomplish this, 25 mL of BC_TO_ stock was added dropwise to 25 mL of CHI stock while stirring continuously. This mixture was then added DCC crosslinker (10 mg). The same procedure was used to prepare the hydrogel with PEI/NO; 25 mL of BC_TO_ stock was added dropwise into 25 mL chitosan stock containing 4 wt% PEI/NO while stirring; crosslinker DCC was used in the same amount (Scheme 1). This crosslinked product was evaporated in 2 cm diameter Teflon petri dishes until a concentrated gel was obtained. The concentrated hydrogel products were frozen at 80 °C for 2 h. The frozen hydrogels were then lyophilized for 6 h at 50 °C in a freeze drier to produce a porous scaffold.

### 2.5. Characterization

#### 2.5.1. Scanning Electron Microscopy (SEM)

The morphologies of BC, BC_TO_, BC_TO_-CHI, and BC_TO_-CHI-PEI/NO were analyzed by field emission electron microscopy using an EBSD system (FE-SEM, Supra 40VP, Carl Zeiss AG, Oberkochen, Germany). The BC-based samples were mounted on carbon tape before being vacuum-coated with platinum for 2 min. The morphology of the test samples was then observed using FE-SEM at acceleration voltages ranging from 1 to 5 kV.

#### 2.5.2. Fourier Transform Infrared Spectroscopy (FTIR)

The FTIR analysis was performed on a Varian^®^ 640 FT-IR Spectrophotometer (Agilent Technologies Varian Inc., California, CA, USA) operating in transmittance mode using the KBr disc method. Forty-eight scans per spectrum were recorded for each sample between 4000 and 500 cm^−1^, with a resolution of 4 cm^−1^. To prepare the KBr discs, KBr and sample were mixed at a ratio of 90:10, and pressed into a pellet under a 10-ton pressing load for 3 min.

### 2.6. Rheological Properties

The rheological properties of BC_TO_-CHI and BC_TO_-CHI-PEI/NO hydrogels were evaluated as previously reported, with some modifications [11]. The steady shear and dynamic viscoelastic properties were measured using a strain-controlled rheometer (Advanced Rheometric Expansion System, Rheometric Scientific, Piscataway, NJ, USA) with a parallel-plate fixture with a radius of 12.5 mm and a gap size of 1.0 mm. All rheological measurements were carried out at a constant temperature of 37 °C across a wide range of strain amplitudes and shear rates. Simulated wound fluid (SWF) was used in this study to cause swelling in hydrogels. Before starting the experiments, a specific volume of SWF was added to 1 g of the test samples under maximum swelling conditions and thoroughly mixed to create a homogeneous hydrogel. All experiments used fresh samples that were rested for 15 min after loading to allow for material relaxation and temperature equilibration. To assess the steady-shear flow behaviors of the test samples, steady rate-sweep tests were performed using a logarithmically increasing scale between shear rates of 1 to 1000 s1. Following that, strain-sweep tests were carried out to identify the nonlinear viscoelastic behavior and the linear viscoelastic region over a strain amplitude range of 0.0625–500% at a fixed angular frequency of 10 rad/s.

### 2.7. In Vitro Nitric Oxide Release

A Sievers 280i chemiluminescence NO analyzer (NOA; Boulder, CO, USA) was used to measure the real-time NO release of BC_TO_-CHI-PEI/NO hydrogels in PBS at pH 7.4 and 37 °C. The analyzer was calibrated before the analysis. Briefly, BC_TO_-CHI-PEI/NO (38.16 mg) was added to 50 mL of PBS (pH 7.4), followed by purging with N_2 (g)_ at a flow rate of 70 mL/min. Another N_2 (g)_ flow was supplied to the flask (200 mL/min) to match the NOA collection rate. When the NO levels reached zero, the analysis was sterminated. Total NO release (t[NO]), maximum flux of NO release ([NO]max), half-life (t_1/2_), and duration of NO release (t_d_) were all calculated.

### 2.8. In Vitro Antibacterial Study

Bacterial viability was measured to determine the antibacterial activity of the crosslinked hydrogels by counting the number of colony-forming units (CFU). The bacterial strains used in this study were MRSA (USA300) FPR 3757 (GenBank accession no.: NC-00793), *Pseudomonas aeruginosa* PAO1 (wild-type prototroph) [38], and *S. aureus* RN4220 [39]. Inoculated bacteria were grown overnight at 37 °C to the mid-exponential phase on TSB and LB media. The resulting bacterial suspension was centrifuged at 8000× *g* for 15 min before being adjusted in concentration with sterile PBS. Bacterial suspensions of *Pseudomonas aeruginosa* (2 × 10^7^ CFU/mL), S. aureus (1 × 10^7^ CFU/mL), MRSA (3 × 10^7^ CFU/mL), and mixed bacteria (3 × 10^6^ CFU/mL) were added to TSB and LB, followed by hydrogels (BC_TO_-CHI and BC_TO_-CHI-PEI/NO) to final hydrogel concentrations of 0.13, 0.25, 0.5, and 1 mg/mL in 12-well plates. As a control, a tube containing bacteria in sterile PBS was used. All samples were incubated in a shaking incubator for 24 h at 37 °C.

Bacterial suspensions were diluted from 10^1^ to 10^8^ in PBS to determine bacterial viability via CFU counts. A 200 µL aliquot of each dilution was plated on LB and TSB agar and incubated overnight at 37 °C. The number of viable bacteria at the time of plating was calculated by counting the number of colonies.

### 2.9. In Vitro Cytotoxicity Study

The Korean Cell Line Bank (Seoul, Korea) provided L929 mouse fibroblast cells. Cells were grown to sub-confluency in RPMI containing 10% (*v*/*v*) FBS and antibiotics (100 µg/mL streptomycin sulfate and 100 IU/mL penicillin G sodium). The cells were trypsinized before being suspended in media at a concentration of 5 × 10^4^ cells per well and plated into 96-well plates. The medium from each wall was removed after 48 h of incubation. Fresh media containing crosslinked hydrogels at concentrations of 0.13, 0.25, 0.5, and 1 mg/mL (100 μL) were added. A standard MTT viability assay was performed after 24 h of incubation. Each well received an MTT solution in sterile PBS, and the incubation period was extended to 2 h. Following that, all solutions were removed from the well, and 150 µL of DMSO was added to solubilize the crystals. The absorbance at 540 nm was comparable to the percentage of viable cells in each well. Cell that had not been treated served as controls. The viability of fibroblast cells in the presence of control and crosslinked hydrogels is reported in comparison to the viability of fibroblasts that have not been exposed to crosslinked hydrogels. The viability of fibroblast was calculated using the following equation:

(1) $$\mathrm{Cell}\;\mathrm{viability}\;\left(\%\right)=\frac{Absorbance\;\left(treated\;cells\right)}{Absorbance\;\left(control\;cells\right)}\times 100$$

### 2.10. In Vivo Wound Healing Assay

As indicated in the document PNU-2020-2839, all animal experiments were carried out in line with the regulations of Korean legislation on animal studies, and were approved by the Ethical Scientific Committee of the Pusan National University, Busan, South Korea on 22 December 2020. We used male ICR mice (7–8 weeks old, Samtako Bio Korea, Osan-si, Korea). Avertin was used to anesthetize mice intraperitoneally. To make full-thickness wounds, the dorsal hair was clipped using an electric razor, and the skin was cut out with an 8 mm biopsy punch. Then, to induce infection, a suspension containing 1.0 × 10^8^ mixed bacteria (*S. aureus*, *Pseudomonas aeruginosa*, and MRSA) was inoculated. The freeze-dried hydrogels were added to cold deionized water (maximum swollen condition, 330%) to form a hydrogel, and were topically applied from day 3 post-injury. Tegaderm^®^ and sterile gauze were used to cover each wound. As a control, mice that had not been treated served as a control. Every three days, the gauze was changed. Photographs of the wounds were analyzed with ImageJ software (Version 1.52i, National Institutes of Health, Bethesda, MA, USA, 2018) to calculate wound size reduction using the equation:

(2) $$\mathrm{Wound}\;\mathrm{size}\;\mathrm{reduction}\;\left(\%\right)=\frac{W_{t}}{W_{0}}\times 100$$

where, *W_t_* is the area of the wound at time *t*, and *W*_0_ is the area of the wound at starting time 0.

### 2.11. Reduction of Wound Bacterial Burden

From day 3 to day 12 post-injury, the bacterial burden (bacterial viability) in the wound was evaluated. A sterile swab with PBS was used to sample the bacteria growing on the wound day 3, day 6 and day 12 post-injury and post-polymicrobial induction. The Bacteria were plated on TSB or LB agar for a qualitative and quantitative examination. Wound skin tissues were homogenized and diluted in sterile PBS. Two hundred microliters of each dilution were plated on TSB agar and incubated overnight at 37 °C. At the time of plating, the number of colonies was counted and used to calculate the number of viable bacteria.

### 2.12. Statistical Analysis

GraphPad Prism 5.03 was used for statistical analysis, which included one-way ANOVA and Bonferroni multiple comparison tests, as well as an unpaired *t*-test (GraphPad Software, La Jolla, CA, USA, 2009). In the event of significant *t*-test deviations, nonparametric Mann–Whitney U tests were used to compare the distributions of two unpaired groups. *p* < 0.05 was considered statistically significant. The data are presented as means ± standard deviation.

## 3. Results and Discussion

### 3.1. Morphological Analysis of BC and BC_TO_

In this study, we aimed to prepare an NO-releasing crosslinked hydrogel for the effective treatment of polymicrobial wound infections. Hydrogels have been widely used for treatment of polymicrobial wound infections. Pati et al. showed that CHI/epsilon-poly-L-lysine hydrogels have a significant effect in eradicating polymicrobial biofilms [40]. Mao et al. also reported the excellent antibacterial activity of BC/gelatin/selenium NPs nanocomposite hydrogels against *Escherichia coli* (*E. coli*) and *S. aureus*, as well as against their multidrug-resistant counterparts (i.e., MDR *E. coli*, and MRSA) [41]. The inherent physicochemical properties of both CHI and BC are suitable for wound dressing materials due to their chemical and physical properties that are comparable to the extracellular matrix, allowing cell proliferation and diffusion [42,43]. Therefore, in this study, we also aim to improve the physicochemical properties of BC and seek to combine modified BC with CHI and NO donor-based polymers to create an optimal hydrogel that enables enhanced treatment of polymicrobial-infected wounds.

**Figure 1:**
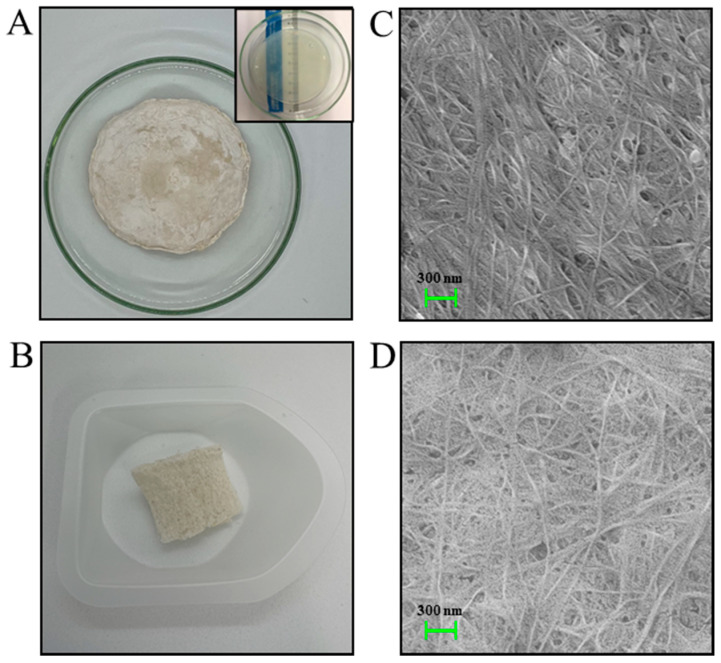
Macroscopic images of (**A**) BC and (**B**) BC_TO_; inset represents wet BC pellicle. Scanning electron microscope (SEM) images of (**C**) BC and (**D**) BC_TO_ (scale bar: 300 nm).

First, we modified the surface of BC by introducing an aldehyde group to BC via TEMPO oxidation. The macroscopic and SEM images of BC and BC_TO_ are shown in Figure 1. Both BC and oxidized BC (BC_TO_) had similar macroscopic morphologies (Figure 1A,B). BC_TO_, however, showed a sponge-like morphology, which is beneficial for use in tissue engineering and drug release. SEM images showed that the internal structure of the nanofibers of BC_TO_ was similar to that of parent the BC (Figure 1C,D). This shows that the introduction of an aldehyde functional group to the BC structure does not affect the overall 3D nanofiber structure of the BC pellicles. Luo et al. reported that TEMPO-mediated oxidation of BC nanofibers does not change the 3D structure of BC, possibly because the oxidation occurs not in ordered packing regions, but in disorder regions, which results in inter-acetal linkages in disorder regions [37].

### 3.2. Morphological Analysis of BC_TO_ and BC_TO_-CHI-PEI/NO Hydrogels

**Figure 2:**
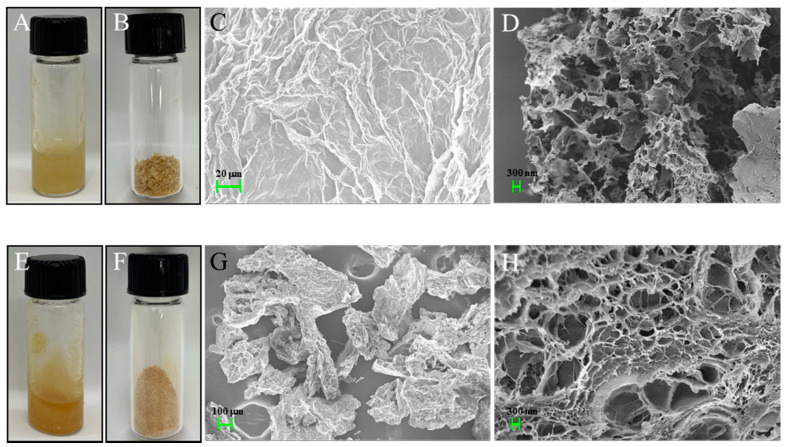
(**A**) BC_TO_-CHI and (**E**) BC_TO_-CHI-PEI/NO hydrogels in simulated wound fluid (SWF). Lyophilized (**B**) BC_TO_-CHI and (**F**) BC_TO_-CHI-PEI/NO. Scanning electron microscopy (SEM) images of (**C**) BC_TO_-CHI and (**G**) BC_TO_-CHI-PEI/NO hydrogels. (**D**) SEM images of a cross-section of the BC_TO_-CHI and (**H**) BC_TO_-CHI-PEI/NO.

The physical appearance and microscopic morphologies of the BC_TO_-CHI and BC_TO_-CHI-PEI/NO hydrogels are shown in Figure 2. Both crosslinked hydrogels showed favorable hydrogel viscosity for cutaneous wound application (Figure 2A,E). The freeze-dried hydrogels confirmed the stability of the hydrogels and NO-loaded hydrogels for long-term storage (Figure 2B,F). The SEM images, including the cross-section images of BC_TO_-CHI and BC_TO_-CHI-PEI/NO, show the fibers and porous structure of the crosslinked hydrogels (Figure 2C–H). In this study, the hydrogels were lyophilized to prevent NO loss during storage. This method also confirms the high NO loading of BC_TO_-CHI-PEI/NO shown in Table 1. The amount of NO in BC_TO_-CHI-PEI/NO was 2.54 μmoles/mg hydrogels. Another possible reason behind high NO loading in BC_TO_-CHI-PEI/NO hydrogels is due to the nanofibrous and porous structure of the prepared hydrogels, which provides a large surface area to accommodate cargo (for example, drugs or cells) into the crosslinked structure of the composite polymers. The pore size of BC_TO_-CHI and BC_TO_-CHI-PEI/NO were 624.68 ± 112.50 nm and 497.54 ± 114.95 nm, respectively.

### 3.3. FTIR Analysis

**Figure 3:**
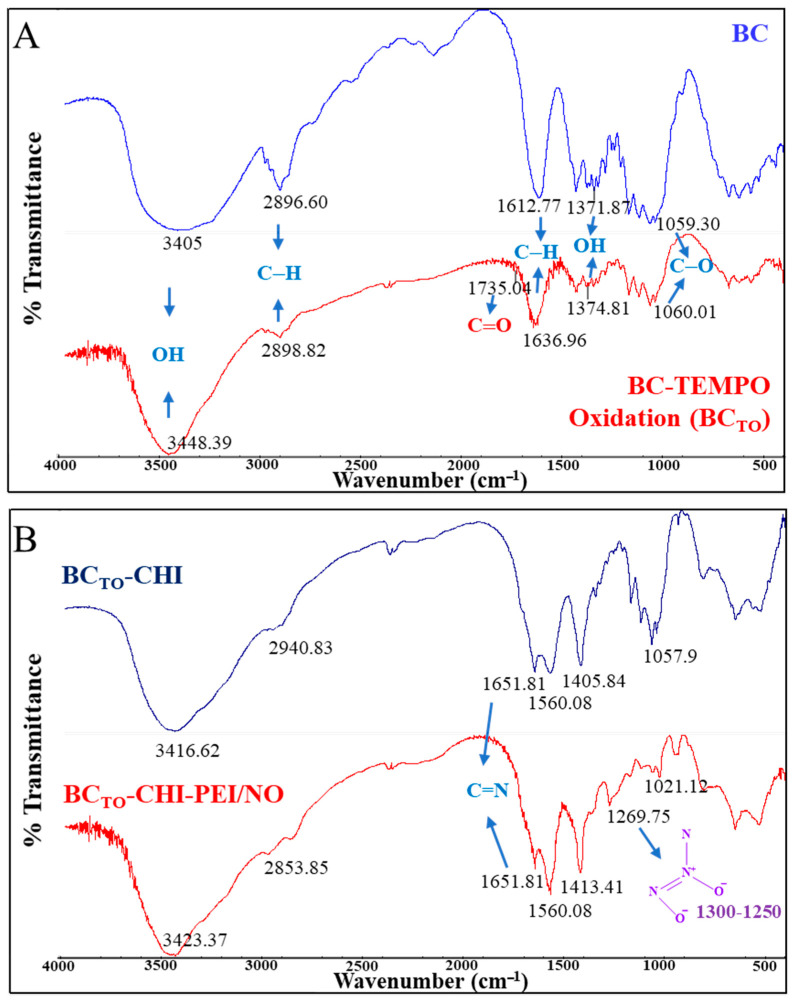
Fourier-transform infrared spectroscopy (FTIR) spectra of (**A**) BC, BC-TEMPO oxidation (BC_TO_); and (**B**) BC_TO_-CHI, BC_TO_-CHI-PEI/NO hydrogels.

Figure 3A shows the FTIR spectra of BC and BC_TO_, whereas Figure 3B shows the spectra of BC_TO_-CHI and BC_TO_-CHI-PEI/NO. The presence of NONOate functional groups in the BC_TO_-CHI-PEI/NO hydrogels was also confirmed by infrared measurement. The BC_TO_-CHI-PEI/NO spectrum had a NONOate peak at 1269.75 cm^−1^ [44], which was missing from the BC_TO_-CHI spectra. The typical infrared spectrum of BC and BC_TO_ has the following absorption bands: the broad bands at 3405 and 3448.39 cm^−1^ (O–H stretching); medium bands at 2896.60 and 2898.82 cm^−1^ (C–H stretching); strong bands at 1612.77 and 1636.96 cm^−1^ (C–H stretching); medium bands at 1371.87 and 1374.81 cm^−1^ (OH stretching); and medium bands at 1059.30 and 1060.01 cm^−1^ (aromatic ester C–O stretching). The peak at 1735.04 cm^−1^ (C=O stretching) confirms the introduction of an aldehyde group via TEMPO reaction in BC_TO_ (Figure 3A). A typical infrared spectrum of BC_TO_-CHI and BC_TO_-CHI-PEI/NO has: the medium bands at 3416.62 and 3423.37 cm^−1^, assigned to the stretching vibration of the OH bond; medium bands at 2940.83 and 2853.85 cm^−1^ (C–H stretching); strong bands at 1651.81 cm^−1^ (C=N stretching); medium bands at 1405.84 and 1413.41 cm^−1^ (OH stretching); and medium bands at 1057.9 and 1021.12 cm^−1^ (aromatic ester C–O stretching) (Figure 3B). The peak at 1651.81 cm^−1^ in both BC_TO_-CHI and BC_TO_-CHI-PEI/NO spectra also confirms the successful crosslinking between the NH_3_^+^ functional group of CHI with COO^−^ of oxidized BC to form an imine (-HC=N-) functional group.

### 3.4. Rheological Properties

**Figure 4:**
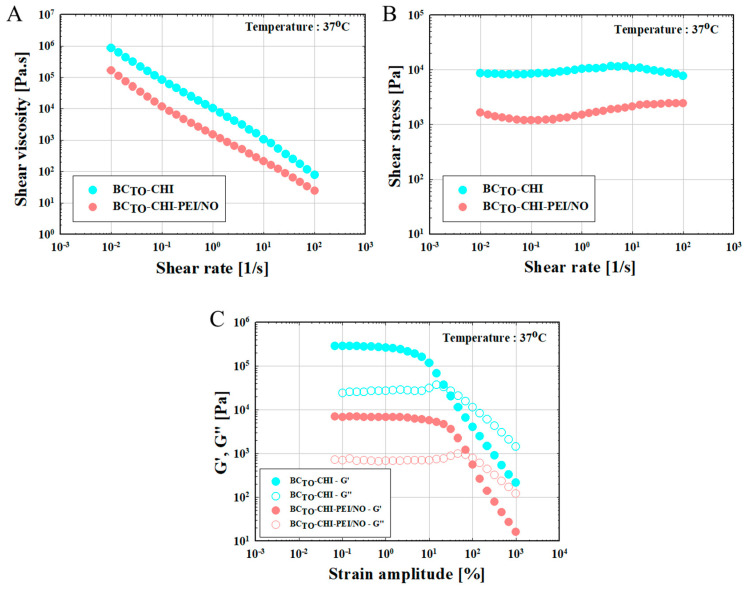
Rheological properties of test samples. (**A**) Shear viscosity of BC_TO_-CHI and BC_TO_-CHI-PEI/NO hydrogels following simulated wound fluid (SWF) absorption at the maximum swollen condition per weight as a function of the shear rate. (**B**) Shear stress vs. shear rate. (**C**) Storage modulus (*G′*) and loss modulus (*G″*) of BC_TO_-CHI and BC_TO_-CHI-PEI/NO hydrogels at 220% swollen condition as a function of the strain amplitude.

The rheological profiles of the test samples are shown in Figure 4. The shear viscosity value is shown as a blank hydrogel compared to that of drug-loaded hydrogels. Both samples had high viscosity values. In particular, the graph shows that the shear stress of blank hydrogels is higher than that of drug-loaded hydrogels. Its physical properties are almost the same as that of a solid that has fixing power when attached to the skin. A relatively large amount of force is required for the removal process, including slight adjustment of the position. In particular, the data at a shear rate of 10 (1/s) or higher were not shown, which indicates that the structural breakdown of blank hydrogels occurs after a specific shear rate, and thus, shape maintenance is difficult. Based on this, NO-loaded hydrogels among the samples have high values for both shear viscosity and shear stress at slow flow rates (when attached to the skin, or slightly finely adjusted to it).

Shear thinning with pseudoplastic flow behavior was observed in all samples (Figure 4A). The BC-CHI-based hard inner gel structure had a high shear viscosity when shear rate was low because it resisted flow; however, when shear rate was increased, the internal structure collapsed, resulting in a rapid decrease in resistance and, consequently, a decrease in shear viscosity (Figure 4B). Pseudoplastic materials have a low viscosity when applied, implying that they can spread out relatively smoothly [11]. Thus, BC_TO_-CHI-PEI/NO could maintain a gel-like structure, and resist small shear stresses, such as gravitational force or brushing against a secondary dressing. These qualities are desirable because wound dressings must be able to maintain enough viscosity at the wound site to prevent excessive leakage [45]. Figure 4C illustrates the storage modulus (*G′*) and loss modulus (*G″*) of each hydrogel-based sample as a function of the strain amplitude at the maximum swollen condition at a fixed angular frequency (ω) of 10 rad/s. BC_TO_-CHI-PEI/NO hydrogels had small strain amplitudes and a slightly dominant (liquid-like) *G″* (which was inversely proportional to the BC_TO_-CHI hydrogels); however, when the strain amplitude increased by 10% to 30% due to structural destruction, both G′ and G″ displayed decreasing shear-thinning behavior. The storage elastic modulus, which is a measure of the material’s solid quality, significantly reduced at this point due to the disintegration of the material’s internal network. The more shear-stressed the material’s structure is, the better the material’s liquid behavior. As a result, the BC_TO_-CHI-PEI/NO hydrogel was able to flow freely and be easily removed from the wound site.

### 3.5. In Vitro Drug Release

**Figure 5:**
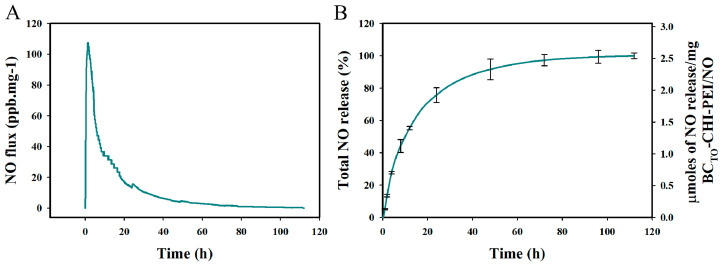
(**A**) Real-time NO release of BC_TO_-CHI-PEI/NO hydrogels. (**B**) Percentage of the total NO released, and amount of NO released over time in phosphate-buffered saline (PBS; pH 7.4, 37 °C), measured using a Sievers 280i chemiluminescence NO analyzer. Data are presented as the mean ± standard deviation; *n* = 3.

We first tested in vitro NO release under physiological conditions (PBS, pH 7.4, 37 °C) before testing the efficiency of crosslinked hydrogels in treating polymicrobial-infected wounds, we first evaluated the. Figure 5A shows a profile of real-time NO release. The maximum flux of NO was 1271 ppb/mg BC_TO_-CHI-PEI/NO hydrogels, with a half-life (t_1/2_) of ~10 h at pH 7.4, 37 °C (Table 1). The BC_TO_-CHI-PEI/NO hydrogels exhibited relatively stable NO release without an initial burst (Figure 5B). They released ~55.25% of NO in the first 12 h, and 75% over 24 h, followed by a gradual sustained release over four days. The sustained release profile of BC_TO_-CHI-PEI/NO can reduce the frequency of BC_TO_-CHI-PEI/NO application to the wound (e.g., every 2–3 days) in order to improve patient compliance, while maintaining the appropriate NO dose. Wynee et al. reported that sternal wound infections and delayed wound healing are associated with the repeated reapplication and removal of dressings [46].

The drug release mechanism from BC_TO_-CHI-PEI/NO is diffusion-controlled. It is the most widely used mechanism to describe the release of drugs (e.g., NO) from hydrogels, where Fick’s law of diffusion with either variable or constant diffusion coefficient is typically used [47]. NO was released from BC_TO_-CHI-PEI/NO in a biphasic pattern. The antibacterial activity of BC_TO_-CHI-PEI/NO (described later in the text) is explained by this release pattern; the initial rapid release kills bacteria and prevents their growth, while the prolonged release helps to maintain an adequate concentration of NO at the site of action.

### 3.6. In Vitro Antibacterial Activity of Hydrogels

**Figure 6:**
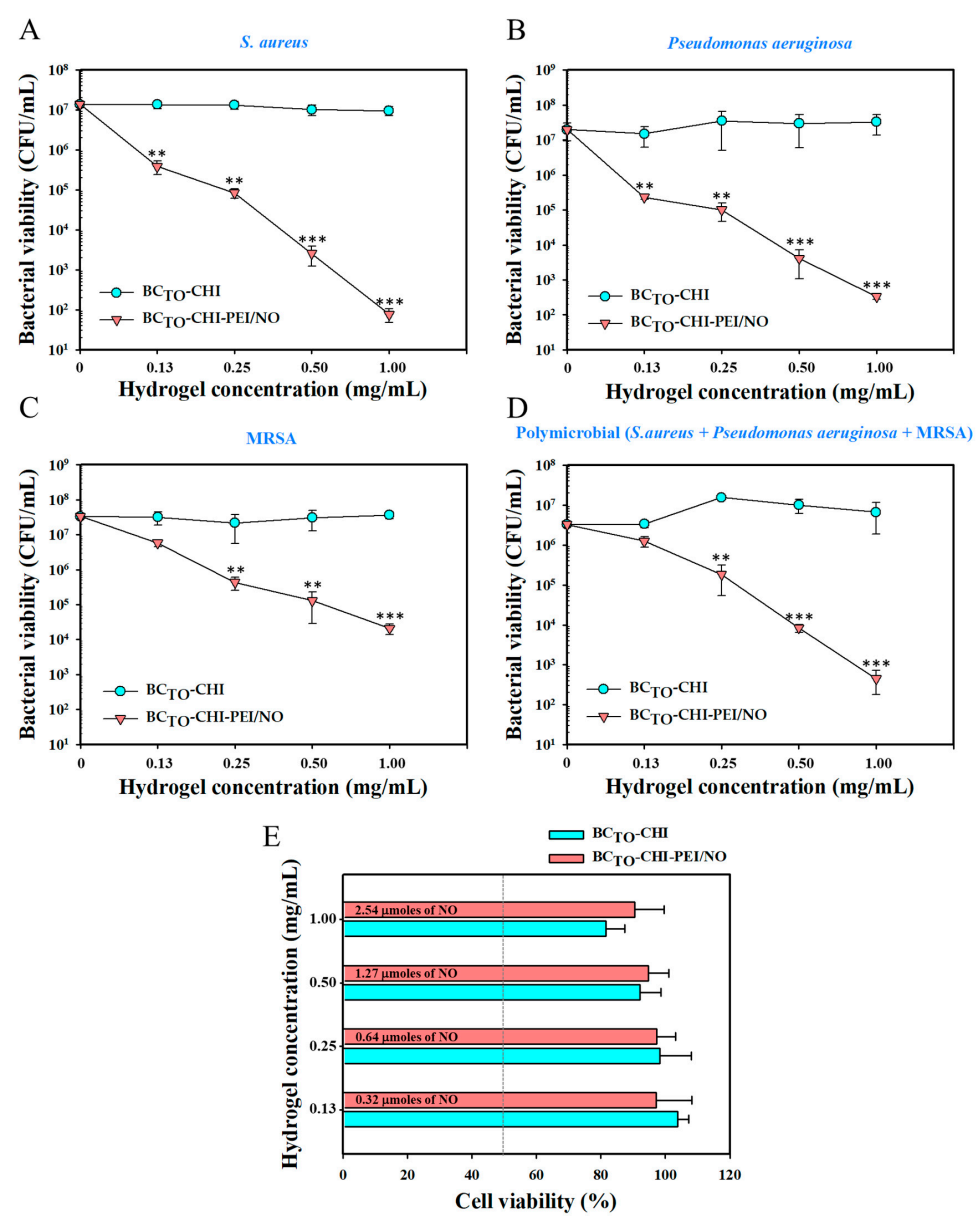
Antibacterial activity of BC_TO_-CHI and BC_TO_-CHI-PEI/NO against (**A**) *S. aureus*, (**B**) *Pseudomonas aeruginosa*, (**C**) MRSA, and (**D**) polymicrobial infection. Data are presented as the mean ± standard deviation; *n* = 3. “**” and “***” indicate *p* < 0.01 and *p* < 0.001 compared to the BC_TO_-CHI, respectively. Viability (%) of L929 mouse fibroblast cells following 24 h of exposure to various concentrations of NPs (*n* = 8) (**E**).

The in vitro antibacterial activity of BC_TO_-CHI and BC_TO_-CHI-PEI/NO against *S. aureus*, *Pseudomonas aeruginosa*, MRSA, and polymicrobial infection (*S. aureus* + *Pseudomonas aeruginosa* + MRSA) was evaluated based on the CFU counts (Figure 6A–D). NO has been known to have antibacterial activity against gram-positive and gram-negative bacteria [48]. Our previously studies on the antibacterial activity of NO delivery systems also support the broad-spectrum antibacterial activity of NO [44,49]. Therefore, in this study, in addition to determining the antibacterial activity of our crosslinked hydrogel against gram-positive (*S. aureus* and MRSA) and gram-negative (*Pseudomonas aeruginosa*) bacteria, we also aimed to determine its activity against polymicrobial infections, because the most likely wounds are polymicrobial infections in the clinical setting.

The results showed that no antibacterial activity against *S. aureus*, *Pseudomonas aeruginosa*, MRSA, and polymicrobial infection was observed with blank hydrogels (BC_TO_-CHI) at different concentrations (0.13, 0.25, 0.5, and 1 mg/mL) after 24 h (Figure 6A–D, cyan circle). In sharp contrast, NO-loaded hydrogels (BC_TO_-CHI-PEI/NO) showed significant antibacterial activity in a concentration-dependent manner against *S. aureus*, *Pseudomonas aeruginosa*, MRSA, and polymicrobial infection (Figure 6A–D, orange triangle). Figure 6A shows that at the lowest BC_TO_-CHI-PEI/NO concentration, *S. aureus* viability was reduced by >1-log (90% killing). The antibacterial activity of BC_TO_-CHI-PEI/NO against *S. aureus* increased with an increasing concentration. At 0.25, 0.5, and 1 mg/mL of BC_TO_-CHI-PEI/NO, bacterial viability was reduced by 2-log (99% killing), >3-log (99.9% killing), and 5-log (99.999% killing), respectively. Figure 6B shows antibacterial activity against *Pseudomonas aeruginosa*. Similarly, the antibacterial activity of BC_TO_-CHI-PEI/NO corresponded with the increase in hydrogel concentration. A reduction of *Pseudomonas aeruginosa* viability by >1-log (90% killing), 2-log (99% killing), >3-log (99.9% killing), and >4-log (99.99% killing) was observed with 0.13, 0.25, 0.5, and 1 mg/mL of BC_TO_-CHI-PEI/NO, respectively (Figure 6B). Antibacterial activity of BC_TO_-CHI-PEI/NO against MRSA, representing antibiotic-resistant bacteria, was also performed (Figure 6C). Approximately more than 2-log (99% killing) and 3-log reduction (99.9% killing) of MRSA were observed at BC_TO_-CHI-PEI/NO concentrations of 0.5 and 1 mg/mL, respectively, whereas at concentrations of 0.13 and 0.25 mg/mL, BC_TO_-CHI-PEI/NO reduced the bacterial viability by ~90% and 99%, respectively. Furthermore, we determined the antibacterial activity of BC_TO_-CHI-PEI/NO against polymicrobial infection, as the bacteria (*S. aureus* + *Pseudomonas aeruginosa* + MRSA) were mixed (Figure 6D). At the lowest BC_TO_-CHI-PEI/NO concentration (0.13 mg/mL), bacterial viability was slightly reduced. At a concentration of 0.25 mg/mL, BC_TO_-CHI-PEI/NO reduced the number of viable cells by >1-log (90% killing). At 0.5 mg/mL of BC_TO_-CHI-PEI/NO, there was a >2-log reduction in bacterial viability (99% killing), which increased significantly to >3-log reduction (99.9% killing) at the concentration of 1 mg/mL.

The contrast of antibacterial activity between BC_TO_-CHI and BC_TO_-CHI-PEI/NO, in which blank hydrogel showed no antibacterial activity against test bacteria even at a high concentration (1 mg/mL), revealed that the NO released from BC_TO_-CHI-PEI/NO is the key factor in eradicating *S. aureus*, *Pseudomonas aeruginosa*, MRSA, and polymicrobial infection. The proposed mechanism of NO as an antimicrobial agent is due to the formation of reactive nitrogen oxide species (RNOS), the byproduct from the reaction of NO (at high concentration) with superoxide or oxygen. This RNOS can directly react with DNA structure, inhibit DNA repair, and increase production of genotoxic hydrogen peroxide and alkylating agents, thus damaging DNA by causing chemical alteration of DNA [50]. Although CHI has been widely known to have broad-spectrum antibacterial activity against gram-positive and gram-negative bacteria [51], the positive charge of CHI was neutralized by crosslinking of the NH_3_^+^ group of CHI with COO^−^ of oxidized BC. This would affect the antibacterial activity of CHI since the most well-known mechanism of CHI in killing bacteria is that the positively charged amino groups of CHI bind to the negatively charged bacterial cell wall, causing disruption, and thus, altering the membrane cell permeability [52]. Previously, we also reported that blank CHI film has no significant antibacterial activity against MRSA [14].

### 3.7. In Vitro Cytotoxicity Study

The toxicity of our crosslinked hydrogels was evaluated using an in vitro cytotoxic test. The cytotoxicity of BC_TO_-CHI and BC_TO_-CHI-PEI/NO to L929 mouse fibroblast cells is shown in Figure 6E. The fibroblast cells have been widely used in in vitro cytotoxicity studies, and are considered suitable cell lines, particularly in wound-healing-related studies, due to their important role in the wound healing process (e.g., extracellular matrix development, and epithelial–mesenchymal interaction) [53]. Moreover, it is reported that fibroblasts have a strong resistance toward NO-induced apoptosis and necrosis [54]. Hydrogels exerted no significant cytotoxicity (>80% viability) on L929 fibroblast cells, regardless of type and concentration. Our results are in line with the previously reported study of CHI-based hydrogels cytotoxicity toward fibroblast cells. Li et al. showed that CHI-based photo crosslinking hydrogels exhibited no toxicity toward L929 cell and SW1353 cell growth [55]. Similarly, Mohamad et al. demonstrated that BC/acrylic acid hydrogel did not induce any cytotoxicity toward L929. Interestingly, they also revealed that the slightly lower toxicity of BC-based hydrogels (i.e., 81.15% of L929 cell viability after two days incubation and direct exposure to hydrogels) may be caused by the acidic environmental condition produced by BC with a high carboxylic group content [56]. This can explain the non-toxic property of our crosslinked hydrogels even at the highest concentration.

### 3.8. In Vivo Wound Healing Assay

**Figure 7:**
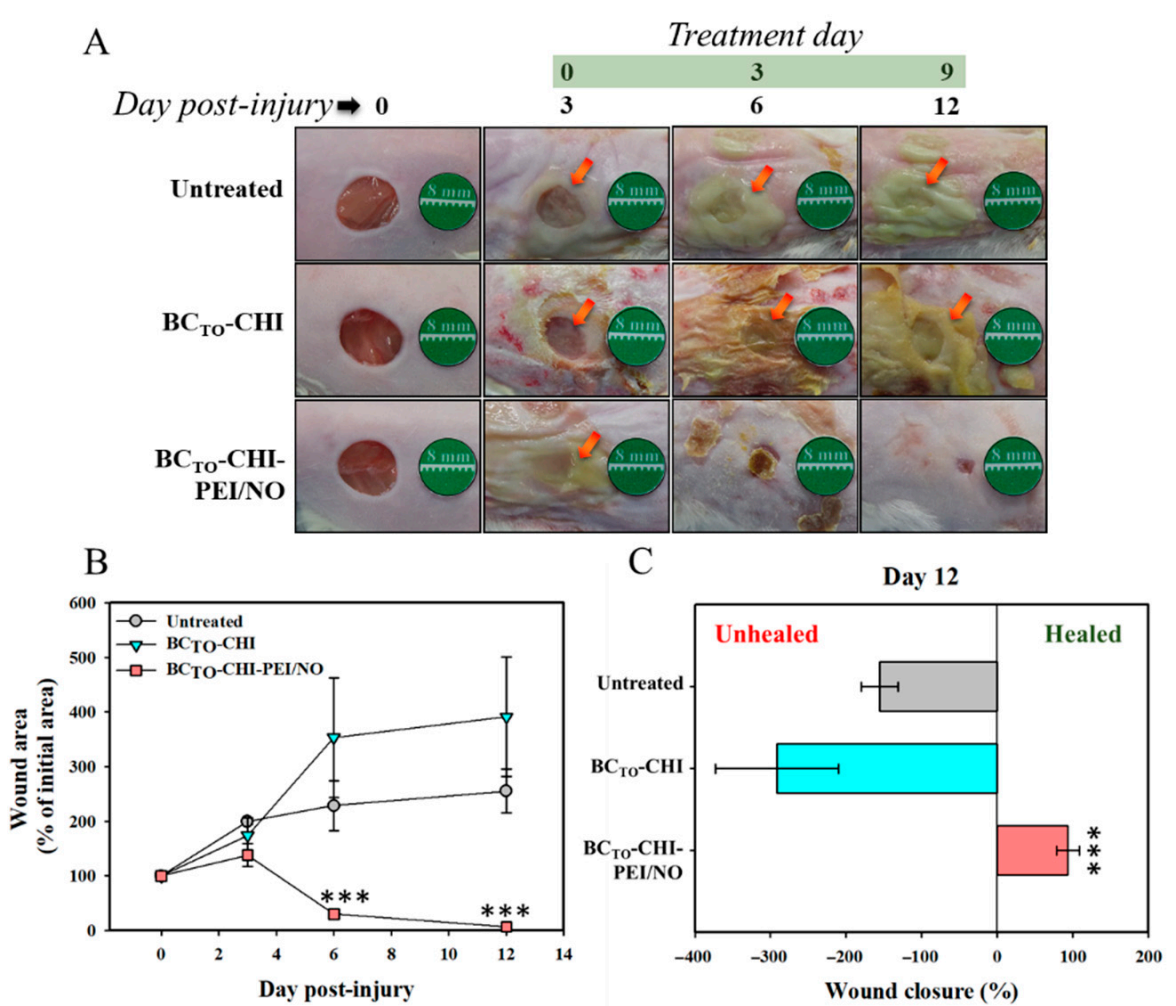
Wound healing assay in mice. (**A**) Representative photographs of polymicrobial-infected wounds of ICR mice treated with or without BC_TO_-CHI and BC_TO_-CHI-PEI/NO hydrogels. The orange arrows indicate polymicrobial infection (**B**) Wound area reduction percentage of mice skin lesions in comparison to the initial 8 mm wound. Values are the mean ± standard deviation, *n* = 10 wounds, and “***” indicates *p* < 0.001 compared to the untreated group. (**C**). Wound closure percentage of mice skin lesions at final day in comparison to the initial 8 mm wound.

An in vivo wound healing assay was carried out to investigate whether BC_TO_-CHI and BC_TO_-CHI-PEI/NO can accelerate the repair of polymicrobial-infected wounds. Wounds were treated with BC_TO_-CHI and BC_TO_-CHI-PEI/NO once every three days based on the NO release profile of BC_TO_-CHI-PEI/NO. Figure 7A shows that BC_TO_-CHI-PEI/NO treatments resulted in lower wound bacterial burdens and faster wound healing, however, untreated and blank hydrogels (BC_TO_-CHI) had no effect on the bacterial burden or the wound size. On day 6 post-injury, the in vivo reduction in bacterial burden was seen in the BC_TO_-CHI-PEI/NO-treated groups. The results confirmed the hypothesis that without eradicating infection, wounds can progress into chronic wounds, which prolongs the inflammation phase, and delays the healing process. Therefore, the uncontrolled bacterial colonization on the wound surface and in adjacent tissue should be prevented by using an antibacterial-based dressing, e.g., NO-releasing BC/CHI crosslinked hydrogels. Accordingly, the BC_TO_-CHI-PEI/NO-treated groups showed wound area reduction from 100% (the initial 8-mm wound) to ~30% (*p* < 0.001) and ~6% (*p* < 0.001) between day 6 and 12 post-injury, respectively (Figure 7B). Moreover, the wound closure (% of initial area) at day 12 post-injury in response to untreated and BC_TO_-CHI treatments was almost complete (93.36%, *p* < 0.001) (Figure 7C). We also found that the BC_TO_-CHI-PEI/NO-treated group showed clear epithelialization with no scab on day 12 post-injury. These findings indicate the potent wound healing activity of NO. It is again worth noting that NO plays a crucial role in several wound healing phases, i.e., as a potent mediator of cell proliferation, angiogenesis, and remodeling [10].

### 3.9. Reduction of Wound Bacterial Burden

**Figure 8:**
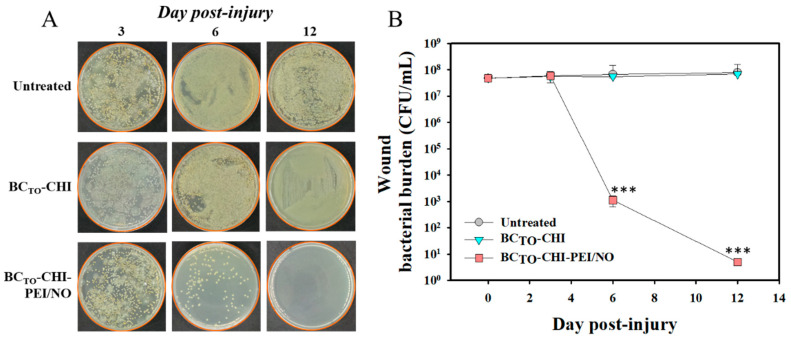
Bacterial burden in the wound. (**A**) Bacterial growth after plating of swab samples and wound tissues on tryptic soy broth (TSB) or Luria-Bertani (LB) agar at day 3, 6, and 12 post-injury. (**B**) Viable counts of bacteria on wounds. Wounds were swabbed, and bacterial burden was examined. Data are presented as the mean ± standard deviation; *n* = 3. “***” indicates *p* < 0.001 compared to the untreated.

Bacterial infection is known to delay wound healing. Therefore, we evaluated the bacterial burden in wounds using viable cell counts. In Figure 8A, the images show multiple bacteria plated out from wound swabs taken on days 3, 6, and 12 post-injury. The untreated and BC_TO_-CHI groups yielded a higher number of polymicrobial colonies compared with the BC_TO_-CHI-PEI/NO groups, which showed the progress of bacterial colony reduction with no bacterial colonies, particularly found on day 12 post-injury. Figure 8B shows that the bacterial burden on wounds of untreated and blank hydrogels (BC_TO_-CHI) groups did not decrease until the final day of the in vivo study. In contrast, the BC_TO_-CHI-PEI/NO-treated groups experienced a >4-log reduction (~99.99% killing) at day 6 post-injury. The BC_TO_-CHI-PEI/NO-treated groups showed a higher level of reduction of bacterial burden, that is >6-log reduction in bacterial viability (~99.9999% killing) at day 12 post-injury.

## 4. Conclusions

In this study, NO-releasing crosslinked hydrogels (BC_TO_-CHI-PEI/NO) were successfully developed. The physical and mechanical properties of BC_TO_-CHI-PEI/NO enable a high NO loading capacity, and improve the rheological properties of the hydrogels. The sustained release of BC_TO_-CHI-PEI/NO over four days is beneficial for the topical administration of the hydrogel to the polymicrobial-infected wounds. The high NO loading of BC_TO_-CHI-PEI/NO enhances the bactericidal efficacy against polymicrobial colonies when compared with BC_TO_-CHI. Moreover, BC_TO_-CHI-PEI/NO significantly accelerates wound healing and re-epithelialization in a mouse model of polymicrobial-infected wounds. We also found that both crosslinked hydrogels are nontoxic to healthy mammalian fibroblast cells. Therefore, the development of NO-releasing hydrogel systems is a promising approach to enhance wound healing, and treat various skin infections.

## Figures and Tables

**Table 1:** NO-release properties (pH 7.4, 37 °C).

	t[NO] ^a^ (μmoles/mg)	[NO]_max_ ^b^ (ppb/mg)	t_1/2_ ^c^ (h)	t_d_ ^d^ (h)
PEI/NO	8.21 ± 1.48	3403.84 ± 646.73	1.53 ± 0.07	11.52 ± 2.19
BC_TO_-CHI-PEI/NO	2.54 ± 0.41	1271.89 ± 267.10	10.03 ± 0.87	111.95 ± 23.51

Values are expressed as the mean ± SD of three measurements. ^a^ Total NO released. ^b^ Maximum flux of NO release. ^c^ NO-release half-life. ^d^ Duration of NO release.
