# Correction: Hasan et al. Nitric Oxide-Releasing Bacterial Cellulose/Chitosan Crosslinked Hydrogels for the Treatment of Polymicrobial Wound Infections. *Pharmaceutics* 2022, *14*, 22

**DOI:** 10.3390/pharmaceutics17091206

**Published:** 2025-09-16

**Authors:** Nurhasni Hasan, Juho Lee, Hye-Jin Ahn, Wook Ryol Hwang, Muhammad Akbar Bahar, Habibie Habibie, Muhammad Nur Amir, Subehan Lallo, Hong-Joo Son, Jin-Wook Yoo

**Affiliations:** 1 College of Pharmacy, Pusan National University, Busan 46241, Republic of Korea; nurhasni.hasan@unhas.ac.id (N.H.); jhlee2350@gmail.com (J.L.); 2 Faculty of Pharmacy, Hasanuddin University, Jl. Perintis Kemerdekaan KM 10, Makassar 90245, Indonesia; 3 School of Mechanical and Aerospace Engineering, Gyeongsang National University, Jinju 52828, Republic of Korea; 4 College of Natural Resources and Life Science/Life and Industry Convergence Research Institute, Pusan National University, Miryang 627706, Republic of Korea

## Error in Figure

In the original publication [1], there was a mistake in Figure 8A as published. Some representative wound bacterial burden images were inadvertently duplicated during figure assembly, leading to repeated panels where different conditions/time points were intended to be depicted. The corrected Figure 8 appears below. The figure legend remains unchanged.

The authors state that the scientific conclusions are unaffected. This correction was approved by the Academic Editor. The original publication has also been updated.

## Figures and Tables

**Figure 8:**
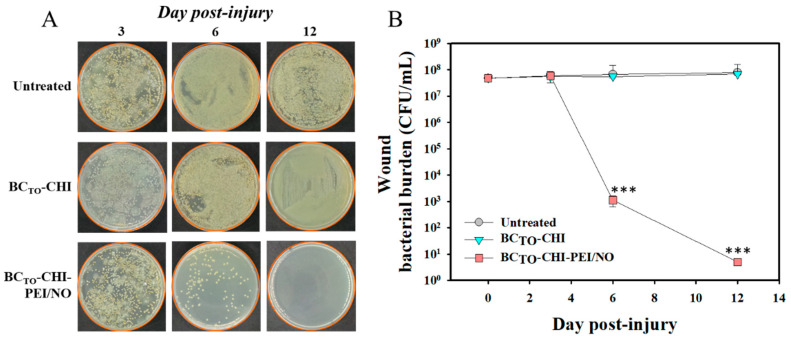
Bacterial burden in the wound. (**A**) Bacterial growth after plating of swab samples and wound tissues on tryptic soy broth (TSB) or Luria-Bertani (LB) agar at day 3, 6, and 12 post-injury. (**B**) Viable counts of bacteria on wounds. Wounds were swabbed, and bacterial burden was examined. Data are presented as the mean ± standard deviation; *n* = 3. “***” indicates *p* < 0.001 compared to the untreated.
